# Frailty level at discharge predicts mortality in older patients with *Clostridioides difficile* more accurately than age or disease severity

**DOI:** 10.1007/s41999-023-00772-3

**Published:** 2023-04-13

**Authors:** Tone Rubak, Simon Mark Dahl Baunwall, Merete Gregersen, Troels Kjærskov Hansen, Jeppe Bakkestrøm Rosenbæk, Lise Tornvig Erikstrup, Christian Lodberg Hvas, Else Marie Skjøde Damsgaard

**Affiliations:** 1 Department of Geriatrics, Aarhus University Hospital, Palle Juul-Jensens Boulevard 99, 8200 Aarhus N, Denmark; 2 Department of Hepatology and Gastroenterology, Aarhus University Hospital, Aarhus, Denmark; 3 Department of Clinical Medicine, Aarhus University, Aarhus, Denmark; 4 Department of Geriatrics, Medical Department, Gødstrup Hospital, Herning, Denmark; 5 Department of Clinical Microbiology, Aarhus University Hospital, Aarhus, Denmark

**Keywords:** *Clostridioides difficile*, Frailty, Mortality, Aged

## Abstract

**Purpose:**

*Clostridioides difficile* infection (CDI) has a high mortality among older patients. Identification of older patients with CDI in increased mortality risk is important to target treatment and thereby reduce mortality. The aim of this study was to investigate mortality rates and compare frailty levels at discharge, measured by the record-based Multidimensional Prognostic Index (MPI), with age and severity of CDI as mortality predictors in patients with CDI diagnosed during hospitalisation.

**Methods:**

This was a population-based cohort study from Central Denmark Region, Denmark, including all patients  ≥ 60 years with a positive CD toxin test without prior infection and diagnosed from 1 January to 31 December 2018. Frailty level, estimated from the electronic medical record, was defined as low, moderate, or severe frailty. CDI severity was graded according to international guidelines. Primary outcome was 90-day mortality.

**Results:**

We included 457 patients with median age 77 years (interquartile range 69–84) and females (49%). Overall, 90-day mortality was 28%, and this was associated with age (hazard ratio (HR): 2.71 (95% confidence interval 1.64–4.47)), CDI severity (HR 4.58 (3.04–6.88)) and frailty (HR 10.15 (4.06–25.36)). Frailty was a better predictor of 90-day mortality than both age (*p* < 0.001) and CDI severity (*p* = 0.04) with a receiver operating characteristic curve area of 77%.

**Conclusion:**

The 90-day mortality among older patients with CDI in a Danish region is 28%. Frailty measured by record-based MPI at discharge outperforms age and disease severity markers in predicting mortality in older patients with CDI.

## Key summary points

**Aim:**

To calculate mortality rates in older patients with *Clostridioides difficile* infection (CDI) and compare multidimensional frailty level, CDI severity and age as predictors of mortality.

**Findings:**

Older patients with their first CDI had a high 90-day mortality of 28%. Multidimensional frailty assessment at discharge outperformed CDI severity and age in predicting 90-day mortality among older patients with CDI.

**Message:**

Compared with age and CDI severity, the multidimensional frailty assessment is the best predictor of 90-day mortality in older patients with CDI.

## Introduction

*Clostridioides difficile* infection (CDI) has a poor prognosis with an average 90-day mortality of 23–46% in selected patient cohorts [1–3]. Mortality rates dramatically increase with age [1, 2, 4]. Population-based mortality rates in older patients with validated CDI are lacking. Preventive strategies and effective therapeutic approaches are warranted to reduce these patients’ risk of dying from CDI [1].

Early identification of patients with a high mortality risk followed by targeted interventions may improve prognosis in patients with CDI. A classification of CDI severity has been suggested [5]. This classification uses clinical and laboratory characteristics assumed to correlate positively with the severity of colitis according to the recommendations of the European Society of Clinical Microbiology and Infectious Diseases (ESCMID) [6]. The classification is in line with the Infectious Diseases Society of America (IDSA) and the Society for Healthcare Epidemiology of America (SHEA) [7], and it is supported by multivariate analyses [8–11]. The established definitions of severe disease have not clearly been associated with mortality rates in the oldest patients, implying that current guidelines need to be further qualified with respect to assessment of older patients with CDI on admission [3].

Frailty is a measure that may encompass the overall health status in patients with CDI. Frailty is a common condition among older adults [12] and develops due to age-related decline in physiological systems, collectively resulting in an increased vulnerability to stressors [13]. Older patients with severe CDI are characterised by a high comorbidity burden, low functional status, high degree of polypharmacy, malnutrition and a need for support in everyday life [2, 13–17]. Collectively, these factors indicate frailty. The literature on the predictive value of frailty among CDI patients is scarce [18] and has not yet been compared with severity of CDI in older patients.

The Multidimensional Prognostic Index (MPI) is a frailty assessment tool with a demonstrated predictive value for mortality in older hospitalised patients [19, 20]. The MPI is based on the Comprehensive Geriatric Assessment (CGA), which allows the clinician to intervene on clinical issues related to the overall health status of the patient [19, 21]. The CGA is a multidisciplinary diagnostic and treatment process identifying medical, psychosocial and functional limitations of a frail older patient [22]. Thus, the frailty-related risk factors for severe CDI among older patients are all included in the MPI [3, 15, 16, 23]. Hence, the MPI may be useful for assessing the risk of mortality in older patients with CDI. A record-based MPI has been validated as a retrospective assessment tool based on medical record data [24].

The aim of the present study was to investigate the mortality rate among older patients with CDI in a Danish region and compare multidimensional frailty level at discharge, CDI severity, and age as predictors of mortality.

## Methods

### Study design and participants

The study was as a population-based cohort study in the Central Denmark Region (CDR), Denmark. Data were collected retrospectively from electronic medical records (EMR). Patients were consecutively included from a complete list of all patients diagnosed with CDI in hospital, outpatient activity and primary health care in the CDR. Patients included in the study were aged  ≥ 60 years and had been diagnosed with an index CDI in the period from 1 January to 31 December 2018. We chose the calendar year 2018 to leave the cohort including the follow-up period untouched by the COVID 19 pandemic. Index CDI was defined as a positive polymerase chain reaction (PCR) test result for *Clostridioides difficile* (CD) toxin A, toxin B or binary toxin, and no previous positive PCR test result for CD toxins or CDI treatment within the previous year. All hospitals in the CDR share the same diagnostic methodology for CD and all analyses were conducted in the same laboratory. The CD toxin PCR test was performed with an in-house PCR, or, for urgent diagnosis, with GeneXpert (Xpert C. difficile BT, Cepheid, Sunnyvale, CA, USA). Any combination of toxins was considered a positive test result. Patients were identified from the Danish Microbiology Database via the national identification number-based Civil Registration Register. The primary outcome was 90-day all-cause mortality from the date of the positive PCR test for index CDI.

### Data collection

EMRs and discharge summaries were reviewed from the date of the index CDI by a specialty registrar in geriatric medicine.

Level of frailty was assessed retrospectively by performing the record-based MPI at discharge from the CDI-related hospital admission [24]. The electronic medical records in the CDR share interdisciplinary information from doctors, nurse, physiotherapy and occupational therapy and includes information from primary healthcare. Assessment of physical functional capacity and social status as well as home care reports are mandatory for documentation. Information is concomitantly documented in the same system. MPI-featured components are described in older inpatients’ EMRs and includes clinical, cognitive, functional, nutritional, and social parameters. The MPI is an aggregated score based on eight items, co-habitation status, number of prescription drugs, Functional Recovery Score Activities of Daily Living (FRS-ADL), Functional Recovery Score Instrumentalized Activities of Daily Living (FRS-IADL), Short Portable Mental Status Questionnaire, Exton Smith Scale, Cumulative Illness Rating Scale-Geriatrics and Mini Nutritional Assessment- Short Form, and is performed in a record-based manner [19, 24]. The MPI sum score is expressed as a number between 0 and 1 by aggregating the total scores of all eight domains and categorised into three groups: MPI-1 (MPI score 0.0–0.33) as low, MPI-2 (MPI score 0.34–0.66) as moderate and MPI-3 (MPI score 0.67–1.0) as severe frailty. When diagnosed with CDI during admission, the record-based MPI was performed as close to the date of discharge as possible but maximally within one week before discharge. Patients diagnosed during outpatient activity or via primary health care were not assessed for frailty, because their EMR data were considered as lacking or insufficient described. The CDI severity score was based on the data recorded in the EMR at the time of the index CDI diagnosis. Data were registered within the CDI-related hospital contact from date of positive PCR test result for CD toxins and up until discharge. Severity of CDI was categorised as mild-to-moderate disease, severe disease and fulminant/complicated CDI [5, 25]. Factors indicating severe CDI were one of the following: P-albumin < 30 g/L, leucocytes > 15 × 10^9/L or abdominal pain. Severe CDI combined with one of the following indicated fulminant CDI: pseudomembranous colitis diagnosed via colonoscopy or sigmoidoscopy; hypotension (defined as systolic blood pressure < 80 mmHg or a need of vasopressor treatment); fever (temperature > 38.5 °C); ileus; change in mental state; admission to an intensive care unit; leucocytes > 35 × 10^9/L or < 2.2 × 10^9/L; lactate > 2.2 mmol/L. Biochemical parameters were collected within 1 week after the positive CD toxin PCR test result. Disease-related symptoms were registered from the EMRs during hospital contacts related to the CDI from date of positive PCR test and up until discharge. For patients diagnosed during outpatient contact, disease-related symptoms were registered within 1 week from date of positive PCR test. Patients diagnosed via primary health care or lacking information in the EMRs during hospital contact were not assessed for CDI severity, because their EMR data were considered insufficient. Study data were collected and managed using the REDCap electronic data capture tool [26].

### Statistical analysis

P-values below 0.05 were considered statistically significant. Patient characteristics were presented as number, mean or median, as appropriate. Mortality rates according to age, CDI severity and frailty were compared in a Cox proportional hazards regression model and displayed using the Kaplan–Meier (KM) survival estimator. The KM analysis assumptions were assessed. The analyses were adjusted for age and sex. Age was adjusted only for sex and stratified into three age groups to compare the hazard ratios (HRs). The proportional hazards assumption was tested with inspection of ‘log–log’ plots. A model control of analysis of severity in three severity levels could not be accepted due to a very limited number of events in the mild severity group. Therefore, we merged the patients with mild and severe CDI and compared them with those with fulminant CDI.

Receiver operating characteristic (ROC) curves were used to quantify the overall ability of age, CDI severity and MPI to discriminate between dead and surviving individuals within 90 days. For the overall measure of the test accuracy, the area under the curve (AUC) was used. A value of  >  0.7 represents a “useful’ predictive ability [27]. To be able to compare the ROC curves for age, severity of CDI and MPI, we only included patients with known severity of CDI and frailty level in the analysis.

Every item of the MPI was tested for association with 90-day mortality in a multivariate logistic regression analysis.

Test for association between recurrence rate within 90 days and age, CDI severity and MPI, respectively, was performed by binary regression analysis adjusted for 90-day mortality.

All analyses were made using Stata version 17 (Stata Corp, Texas, USA).

### Ethics approval

The study was defined as a quality improvement study which, according to Danish law, solely requires approval from the hospital board. This was confirmed by the Central Denmark Region Ethics Committee (1-10-72-1-21). Permission to assess patient EMRs was obtained by the hospital boards at a University Hospital and all regional hospitals in the CDR (12-11-20).

## Results

### Patient characteristics

**Fig. 1:**
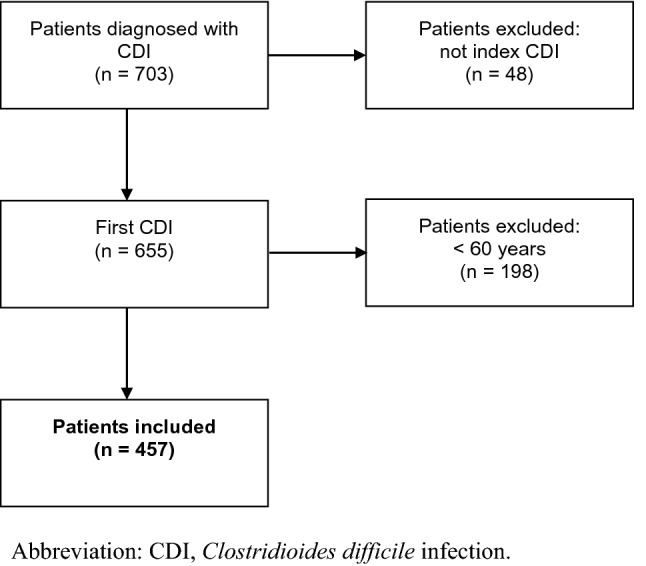
Flow diagram. A total of 703 patients with a positive PCR test for *Clostridioides difficile* in the Central Denmark Region, from 1 January through 31 December 2018, were screened. Patients who had experienced a prior CDI less than one year ago and patients younger than 60 years of age were excluded

**Fig. 2:**
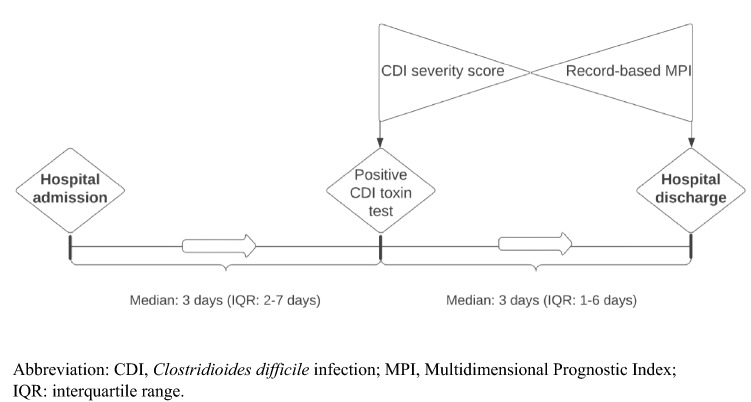
Collection of data, timeline

A total of 457 consecutive patients with their first CDI in 2018 were included (Fig. 1). Estimation of CDI severity was possible in 412 patients and assessment of frailty by record-based MPI in 387 (Table 1). Patients diagnosed during outpatient activity (*n* = 56) or via primary health care (*n* = 14) were not assessed for frailty because their EMR data were missing or insufficient. Median age was 77 years (interquartile range (IQR): 69–84 years), and 49% were females. The majority was classified as having severe (*n* = 189; 41%) or fulminant CDI (*n* = 190; 42%) and as moderately (*n* = 136; 30%) or severely frail (*n* = 171; 37%). Fulminant CDI was diagnosed based on pseudomembranous colitis (*n* = 21; 11%), hypotension (*n* = 54; 28%), fever (*n* = 36; 19%), ileus (*n* = 10; 5%), change in mental state (*n* = 106; 56%), admission to an intensive care unit (*n* = 32; 17%), leucocytes > 35 × 10^9/L (*n* = 18; 9%), leucocytes < 2.2 × 10^9/L (*n* = 10; 5%) and lactate > 2.2 mmol/L (*n* = 43; 23%). Patients fulfilling more than one criteria for fulminant CDI was *n* = 103; 54%. Patients classified as fulminant CDI only because of change in mental state was (*n* = 29; 15%). Most patients (*n* = 387; 85%) were diagnosed with CDI during hospital admission. Median duration from positive PCR test (time of CDI severity score) and until date of discharge (time of multidimensional frailty assessment) was 3 days (IQR: 1–6 days) (Fig. 2).

**Table 1:** Patient characteristics, patients aged ≥ 60 years with *Clostridioides difficile* infection in the Central Denmark Region in the period from 1 January to 31 December 2018

	*n* (%)
Total, *N*	457 (100)
Age groups (years)	
60–69	118 (25)
70–79	158 (35)
80–max	181 (40)
Severity	
Mild	33 (7)
Severe	189 (41)
Fulminant	190 (42)
Unknown	45 (10)
Frailty level	
Low (MPI-1)	80 (18)
Moderate (MPI-2)	136 (30)
Severe (MPI-3)	171 (37)
Unknown	70 (15)
Gender, *n* (%)	
Female	223 (49)
Habitation status	
Living in own home	384 (84)
Nursing home resident	73 (16)
CDI diagnose, site	
Primary healthcare	14 (3)
Outpatients	56 (12)
Inpatients	387 (85)
Primary disease requiring admission	
Clostridioides difficile	86 (19)
Other enteral infectious disease	14 (3)
Urinary tract infection	20 (4)
Sepsis	27 (6)
Erysipelas	3 (1)
Viral infectious disease	4 (1)
Pneumonia	51 (11)
Chronic Obstructive Pulmonary disease	20 (4)
Cardiovascular disease	13 (3)
Endocrine disease	6 (1)
Dehydration and electrolyte disturbance	14 (3)
Gastrointestinal disease	31 (7)
Renal disease	9 (2)
Rheumatic disease	12 (3)
Anaemia	3 (1)
Central nervous system disease	5 (1)
Delirium	2 (0,5)
Other skin diseases	2 (0,5)
Lesions and intoxication	20 (4)
Cancer	15 (3)
Other	30 (7)
Unknown	70 (15)
Toxin profile	
Toxin A/B	455 (99)
Binary toxin	75 (16)
Subtype 027	2 (0.4)
CDI definition^a^	
Healthcare facility-onset (HO)	193 (42)
Community-onset, healthcare facility-associated (COHCFA)	137 (30)
Community-associated (CA)	127 (28)
Treatment at index CDI, *n* (%)	
No treatment	55 (12)
Metronidazole	257 (56)
Vancomycin	69 (15)
Metronidazole + Vancomycin	69 (15)
Vancomycin + Fidaxomicin	3 (0.7)
Vancomycin + Feacal microbiota transplantation	4 (0.9)

*CDI Clostridioides difficile* infection, *MPI* multidimensional prognostic index

^a^HO: case of CDI collected > 3 days after admission to facility; CO-HCFA: case of CDI occurring within 28 days after discharge from a healthcare facility; CA: case of CDI occurring more than 28 days after discharge from a healthcare facility

### Mortality and recurrence

**Fig. 3:**
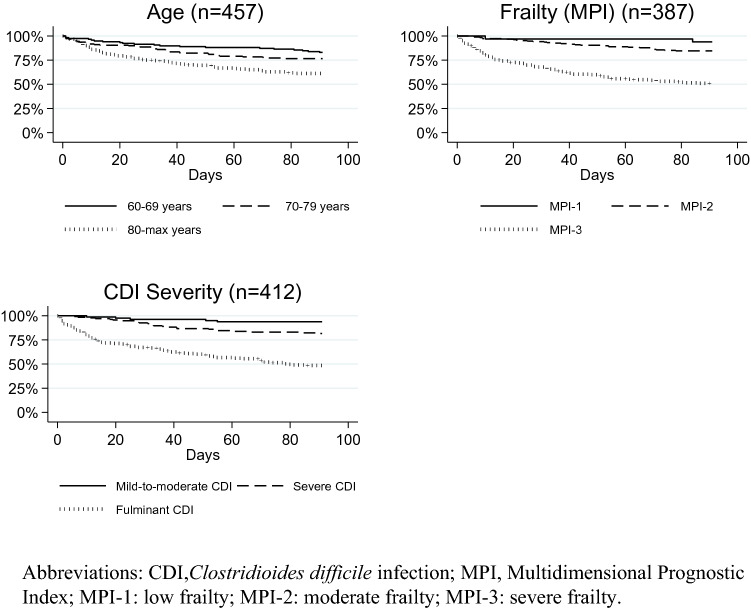
Kaplan–Meier survival curves for age, severity and frailty

Within 90 days following the initial diagnosis, 127 (28%) of all 457 patients had died. Among patients with severe frailty (MPI-3), 88 (51%) of 171 had died. The overall 30-day mortality was 16%. Patients aged 80 years or older had a higher 90-mortality than patients younger than 70 years (HR_adjusted_ = 2.71 (95% CI 1.64–4.47)) (Table 2). Increasing age reduced day-to-day survival, especially for patients aged 80 years or older (Fig. 3).

**Table 2:** 90-Day mortality and hazard ratio estimates for age, severity and frailty of *Clostridioides difficile* infection

	Total, *n* (%)	Death within 90 days, *n* (%)	Crude HR (95% CI)	*P*-value	Adjusted HR (95% CI)^a^	*P*-value
Total	*N* = 457	127 (28)	–	–	–	–
Age groups (years)						
60–69	118 (25)	20/118 (17)	1.00 (reference)	–	1.00 (reference)	–
70–79	158 (35)	37/158 (23)	1.46 (0.85–2.51)	0.175	1.48 (0.86–2.55)	0.162
80-max	181 (40)	70/181 (39)	2.67 (1.62–4.38)	< 0.001	2.71 (1.64–4.47)	< 0.001
Severity (*N* = 412)						
Severe^b^	222 (54)	31/222 (14)	1.00 (reference)	–	1.00 (reference)	–
Fulminant	190 (46)	94/190 (49)	4.69 (3.12–7.05)	< 0.001	4.58 (3.04–6.88)	< 0.001
Frailty level (*N* = 387)						
Low (MPI-1)	80 (18)	5/80 (6)	1.00 (reference)	–	1.00 (reference)	–
Moderate (MPI-2)	136 (30)	25/136 (18)	3.10 (1.19–8.10)	0.021	2.70 (1.03–7.11)	0.044
Severe (MPI-3)	171 (37)	88/171 (51)	11.32 (4.60–27.90)	< 0.001	10.15 (4.06–25.36)	< 0.001
Unknown	70 (15)	9/70 (13)	–	–	–	–

Patients with unknown MPI or CDI severity were not included in the analysis, but separate sensitivity analyses were made

*CDI Clostridioides difficile* infection, *MPI* multidimensional prognostic index

^a^Adjusted for age and gender. Age groups only adjusted for gender

^b^Mild (*n* = 33) and severe (*n* = 189) CDI were merged

Ninety-day survival according to CDI severity showed a reduced day-to-day survival, especially in patients with fulminant CDI, whereas nearly all patients with mild CDI survived through 90 days (Fig. 3). Patients with fulminant CDI had a higher day-to-day mortality hazard than patients with severe CDI, as reflected in the adjusted HR 4.58 (95% CI 3.04–6.88).

An increasing frailty level reduced day-to-day survival (Fig. 3). The 90-day mortality risk differed significantly between the MPI groups (Table 2). In patients with severe frailty (MPI-3), the adjusted HR was 10.15 (95% CI 4.06–25.36) compared with patients with low frailty (MPI-1). For patients with moderate frailty (MPI-2), the adjusted HR was 2.70 (95% CI 1.03–7.11) as compared with MPI-1.

All the eight items of the MPI were associated with 90-day mortality except number of prescription drugs.

In addition to mortality, we examined CDI recurrence. Overall, 107 (23%) of all 457 patients had CDI recurrence within 90 days from date of positive PCR toxin test. No patients had a colectomy. There was no statistically significant association between recurrence of CDI and age (*p* = 0.776), CDI severity (*p* = 0.906) or MPI (*p* = 0.491).

### Comparison of predictors of 90-day mortality

**Fig. 4:**
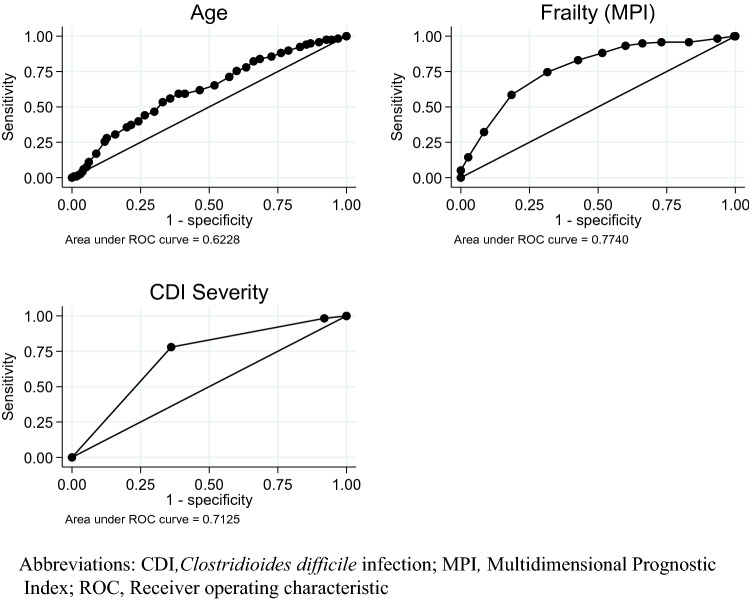
ROC curve estimates for 90-day mortality for age, CDI severity and frailty measured by MPI. Only patients with a known severity and frailty level (*n* = 378) were included in the analysis

The areas under the ROC curves were used to quantify the overall ability of age, CDI severity and MPI to predict 90-day mortality (Fig. 4). The discrimination of the MPI according to 90-day mortality was good with a ROC area of 77%, which differed statistically significant from the ROC area for discrimination of age (*p* < 0.001) and CDI severity (*p* = 0.04). The discrimination of CDI severity according to mortality was 71% and differed significantly only when compared with the ROC area for discrimination of age (*p* = 0.017).

### Sensitivity analysis

To test the robustness of risk estimates and investigate whether missing data may have had an impact on the adjusted HRs, we performed a sensitivity analysis. Insufficient information in the EMRs to calculate a CDI severity score (n = 45 (10%)) and frailty level (*n* = 70 (15%)) was stated as missing values.

When patients with an unknown level of severity were coded as mild CDI and included in the analysis, the adjusted HR was 2.80 (95% CI 0.98–7.99) for severe disease compared with mild CDI. For fulminant disease, the adjusted HR was 11.73 (95% CI 4.29–32.04) compared with mild CDI. Thus, including patients with an unknown level of severity in the analyses did not change the results.

When patients with an unknown frailty grade were merged with MPI-1 and included in a sensitivity analysis, the adjusted HRs remained statistically significant only for MPI-3 (adjusted HR = 6.76 (95% CI 3.78–12.10)) when compared with the merged group. When MPI-1 was merged with MPI-2, the adjusted HR for MPI-3 remained significant (adjusted HR = 4.76 (95% CI 3.11–7.30)).

## Discussion

The key finding of this population-based study was a 90-day mortality of 28% in patients with CDI and older than 60 years. Among patients with severe frailty 51% died before 90 days. Both patient age, disease severity, and frailty level at discharge predicted mortality. Frailty level outperformed age and severity in predicting mortality at 90 days.

The finding of a high 90-day mortality is in accordance with previous studies [1, 2, 4, 28]. The present study provides population-based mortality data and included all patients with a positive CDI toxin test during hospital admission as well as outpatient treatment and primary healthcare, with a record-based validation of clinical CDI. Our study thus describes the overall mortality in age-specific groups of patients with CDI in a Danish region and not merely in a selected cohort.

Literature on multidimensional assessed frailty in patients with CDI is limited. In a retrospective observational study including 33 patients older than 75 years [18], most of the patients (84%) were severely frail according to the MPI, but no statistically significant difference with respect to mortality was identified. This may be explained by a low number of participants and because only patients admitted to a geriatric care unit were included, whereas we included all patients presenting with a positive CDI test, regardless whether they came from a geriatric or other medical departments or surgical departments. The MPI was performed during the first 48 h after admission whereas we performed the MPI at discharge. The predictive value of MPI for patients with CDI at discharge was in line with its value in other medical inpatients at time of discharge, showing an AUC for 90-day mortality of 0.76 [29]. The patients were older (≥ 75 years) and required daily assistance or had a Charlson Comorbidity Index  ≥ 1, whereas patients in our study were included solely if they had a positive CDI test and were aged  ≥ 60 years. This indicated that frailty may play a crucial role for patients with CDI. Frailty rating may be performed in several ways. We suggest that the MPI is relevant for the evaluation of patients with CDI because it includes the CGA-based aspect. A concomitant clinical intervention using the CGA approach to issues identified via the MPI may lead to less severe outcomes and ultimately fewer deaths. The role of CGA employed as an intervention in old patients with CDI needs to be further investigated.

Time of frailty assessment can be discussed. In this study we chose to assess frailty measured by the record-based MPI at discharge, as this timing made the retrospective assessment based on information from the EMRs as precise as possible. The aggregate MPI score was a better predictor of mortality than the individual MPI items. Except for number of prescription drugs all the MPI items were associated with 90-day mortality. However, what would also be interesting is to know the baseline frailty status and the outcome of CDI. However, such study design would be very challenging and resource intensive. We chose the record-based MPI as a marker of frailty recognizing that the measurement may be affected by the state of acute illness. Therefore, the MPI at discharge may merely reflect the acute deconditioning due to the disease process and CDI and not the true frailty status as the baseline frailty status is unknown. However, risk assessment is only meaningful at the time of CDI to help the clinician evaluate the patient, including the choice and timing of treatment.

Frailty had a higher predictive value of 90-day mortality than disease severity. Severity classification which is based on clinical and biochemical parameters that may not independently capture the degree of CDI severity in older patients. Studies conducted in similar-aged populations have focused on individual risk factors for severe outcomes such as level of comorbidity, nutritional and functional status [3, 16, 23]. These factors are included in the MPI but not in the classification of CDI severity. Furthermore, in frail old patients, typical illness symptoms are often absent [30] or atypical [31]. Instead, patients may present with symptoms such as general weakness and immobility, commonly known as the “geriatric syndrome” [32], which may be better assessed using a multidimensional frailty approach. The population of this cohort was older and, as frailty increases steadily with age, the prevalence of frailty among our cohort was presumably larger than in a younger patient cohort with CDI [33]. The higher predictive value of 90-day mortality for multidimensional frailty when compared to CDI severity may be because the components of the record-based MPI and CDI severity classification differ.

Disease severity, although outperformed by frailty level, also predicted mortality at 90 days. Our study thus confirms the clinical applicability of the severity classification. Yet, the use of CDI severity markers in older patients has been questioned. In a study of patients older than 65 years, the accepted CDI severity criteria (IDSA) were not significantly associated with poor outcomes [34]. Similarly, a cohort study of patients older than 80 years found no significant difference in 30-day mortality between patients defined as having severe or non-severe CDI according to the IDSA definition of severe CDI [3]. Other studies indicate that comorbidity and not the IDSA severity score is a key driver for mortality [35, 36]. However, in the present study, the CDI severity level [25] was validated and it had a significant predictive ability to predict 90-day mortality. This discrepancy between the results of previous studies and our study may be explained by the fact that previous studies [3, 34] only examined risk of death based on laboratory parameters following the IDSA and did not include clinical symptoms. The adequacy of the European markers of the risk of severity was reported in a meta-analysis [37]. In accordance with our findings, in a cohort study of patients older than 75 years, the European markers were shown to be adequate in predicting mortality in patients  ≥  75 years; and the authors concluded that level of severity was a risk factor of mortality [15]. Because of varying definitions of standard severity markers, it is difficult to compare data between studies. Our severity assessment differed from the studies mentioned above as we included abdominal pain and not age above 65 years or a rise in creatinine level in the CDI severity assessment. In the daily clinical work, level of CDI severity heightens the clinician’s awareness of the importance of the CDI among older patients. Severe and fulminant CDI should impose on clinicians not to hesitate initiating treatment to prevent death.

High age is a risk factor for severe CDI and complicated CDI outcomes, including death [38, 39]. In accordance with our findings, previous studies of adult CDI patients of all ages reported that age older than 80 years is a significant risk factor for death [40, 41]. In a study including patients aged  ≥  18 years [40], age was a better predictor of death during hospitalisation than in our study. The older population in our cohort may explain this difference. Compared with the record-based MPI, the predictive ability of age for mortality was minor. This indicates that age alone is insufficient as a prognostic factor in the older population when assessing the overall severity of CDI.

It is striking that metronidazole was the most frequent initial treatment and vancomycin less frequently prescribed considering the severity of the disease. This implies suboptimal treatment and lack of adherence to guidelines at that time [7, 42]. This may explain the high relapse rates. No colectomy was performed although 42% of the patients had fulminant CDI. This may indicate a misclassification bias or suboptimal management of the very severe cases. In terms of severity classification, it is a limitation to the study that it is not clear whether the hypotension and lactic academia were due to CDI or the primary disease process warranting admission. Emergency colectomy is indicated for a small minority of patients with severe CDI who have the potential to recover from major surgery [43, 44]. However, the mortality rate following surgery remains extremely high and the timing of such surgery is poorly defined [45, 46]. Meta-analysis of high-quality studies revealed that the strongest predictors of postoperative death were those relating to preoperative physiological status [47]. We propose that the patients in this cohort were too frail to be candidates for colectomy. The clinical treatment practice documented in our data was suboptimal suggesting a delayed implementation of the guidelines. This delay may be pronounced among older patients and is, therefore, an independent learning from our study.

The strengths of our study include a large sample size and a complete description of mortality of all CDI patients with a positive CD test during the period. This is a population-based study which includes all CDI patients with a positive CD test during the period, disregarding if patients are diagnosed in primary health care or in hospital. Patients were included based on age and positive PCR test solely, minimising the risk of selection bias. No patients were lost to follow-up on the primary outcome. Providing the MPI domains allowed us to conduct a multidimensional characterisation of inpatients with CDI. This may be used to design a multidimensional approach to patients with CDI in future studies.

Our study has important limitations. Firstly, data were collected retrospectively and rely on the completeness of the medical records which may induce information bias and underestimate the frailty level. The prognostic ability of the record-based MPI has recently been validated in a Danish cohort study including 1190 older (75 +) medical patients admitted to a general internal medicine unit [29] It is a limitation that the record-based MPI has not been evaluated specifically in surgical departments. However, at admission, all Danish EMRs encompass updated information from primary health care as well as interdisciplinary observations. The municipality delivers data regarding need for assistive remedies and allocation of home care. Medication charts from primary care are linked to the medical records medication list. Therefore, we consider the data from the EMRs sufficient for a record-based MPI measurement.

Diagnosing CD necessitates both clinical evidence of diarrhoea and evidence of toxinogenic CD. A study limitation is the retrospective collection of clinical data from the EMRs. All patients were diagnosed with acute onset diarrhoea and tested for CDI based on clinical suspicion. In standard clinical practice, the laboratory rejects faecal specimens if they are not liquidous (e.g., Bristol stool chart score 6–7), as determined by visual inspection. The use of a standalone PCR test for CDI diagnosis is debated because of the high prevalence of asymptomatic carriers with CD [48]. Although asymptomatic carriage may affect the results of this study, limiting the inclusion criteria to patients with onset and relevant exposure may mitigate its impact, as all patients exhibited new-onset diarrhoea.

Secondly, frailty data were collected only for patients admitted to hospitals where medical records were available. The role of frailty as a predictor of mortality among patients with community-acquired CDI, therefore, remains unknown. The low number of community-acquired CDI may be explained by a low awareness of CDI in the primary healthcare setting. Because the number of community-acquired CDI cases was limited, we believe that the record-based MPI provided a valuable tool to assess frailty in older patients with CDI. Thirdly, the severity definition includes components collected at the time of the positive PCR test whereas the frailty measure is more closely related to the discharge date. Because the median duration from date of positive PCR test and until date of discharge of CDI-related admission was 3 days, any bias resulting from this difference is considered low. It was surprising that more than 40% of the cases were classified as fulminant CDI. Fifteen percent was classified as such solely due to a change in mental status which might have overestimated the prevalence of patients with fulminant CDI. On the other hand, we may consider whether frail older patients may present with atypical symptoms [30, 31] which may challenge the assessment of severity of CDI. Traditional severity indices for CDI are not necessarily present in older patients [3]. A change of mental status may be an important indicator of fulminant CDI in older patients. If not recognized early this may delay initiation of appropriate treatment and thereby increase the risk of an exacerbation of prognosis [49]. Causes of death were not recorded because it is difficult to determine cause-specific deaths from CDI and the degree to which CDI contributes to death.

Finally, current mortality rates might differ from the rates in this study since practice has changed in CDI management the last few years. In a Danish setting, vancomycin is considered first choice and faecal microbiota transplantation (FMT) has emerged as a life-saving treatment in patients with first and recurrent CDI in achieving sustained resolution from CDI [25, 50, 51]. FMT treatment tends to reduce mortality compared with treatment with vancomycin [52]. The 90-day mortality rate may be reduced with current antibiotic approaches combined with faecal microbiota transplantation.

In conclusion, in a population-based, well-defined, and consecutively included cohort of older patients with their first CDI, we found a high 90-day mortality, increasing with age, disease severity and frailty level. Multidimensional frailty measured by the record-based MPI was a superior predictor of mortality in patients with CDI, outperforming both age and CDI severity. An imminent need exists for future studies to investigate the effect of an early frailty assessment with CGA on CDI outcomes to optimise the clinical management of older patients with CDI.

## Data Availability

Code book for data entry databases and all other project-related raw material are available upon reasonable request to the corresponding author.
